# Propolis Varnish: Antimicrobial Properties against Cariogenic Bacteria, Cytotoxicity, and Sustained-Release Profile

**DOI:** 10.1155/2014/348647

**Published:** 2014-05-13

**Authors:** Mariana P. De Luca, Juçara R. Franca, Filipe Augusto F. F. Macedo, Liliana Grenho, Maria Esperanza Cortes, André Augusto G. Faraco, Allyson N. Moreira, Vagner R. Santos

**Affiliations:** ^1^Department of Paediatric Dentistry and Orthodontics, Faculty of Dentistry, Federal University of Minas Gerais, Campus Pampulha, Avenida Presidente Antônio Carlos 6627, 31.270-901 Belo Horizonte, MG, Brazil; ^2^Department of Pharmaceutical Products, Faculty of Pharmacy, Federal University of Minas Gerais, Campus Pampulha, Avenida Presidente Antônio Carlos 6627, 31.270-901 Belo Horizonte, MG, Brazil; ^3^Department of Restorative Dentistry, Faculty of Dentistry, Federal University of Minas Gerais, Campus Pampulha, Avenida Presidente Antônio Carlos 6627, 31.270-901 Belo Horizonte, MG, Brazil; ^4^Biomedical Engineering Institute (INEB), Porto University and Faculty of Engineering, DEMM, Porto University, Porto, Portugal; ^5^Department of Oral Surgery and Pathology, Faculty of Dentistry, Federal University of Minas Gerais, Campus Pampulha, Avenida Presidente Antônio Carlos 6627, 31.270-901 Belo Horizonte, MG, Brazil

## Abstract

Varnishes are preparations that differ in the polymeric matrix and therapeutical agents. In dentistry they are used to prevent caries. In this study we developed a propolis varnish, considering propolis properties against cariogenic bacteria. To a chitosan polymeric base (CHV) was added ethanolic propolis extract in different concentrations: PV1 (5%), PV2 (10%), and PV3 (15%). Antimicrobial activity was carried out against *Streptococcus mutans* (SM), *Streptococcus sanguinis* (SG), *Streptococcus salivarius *(SS), and *Lactobacillus casei* (LC) through agar diffusion method. The three propolis concentrations incorporated were effective in inhibiting the growth of all microorganisms, but without significant difference between the zones of inhibition observed. Cytotoxicity assay was done by MTT method. Data were analyzed by one-way ANOVA and Bonferroni test. None of the varnishes were cytotoxic, keeping 80% of viable cells, while CHV allowed cellular proliferation (120%). Sustained-release test was carried out by applying 40 **μ**L of each varnish in the buccal surface of bovine teeth and kept in an ethanol/water solution removed in regular times. According to the “independent model approach,” the release profiles were distinct from each varnish and the most prolonged was PV3 (8 weeks). Varnish formulations had satisfactory antimicrobial activity against cariogenic bacteria and have a low cytotoxicity (<50%).

## 1. Introduction

Dental caries is among the most prevalent chronic human infectious diseases affecting children and adults worldwide [1, 2]. One of the mechanisms that allow the control of caries is the decrease of dental biofilms, which is a complex hole of microorganisms and metabolic products fixed in a polymeric matrix adhered to the tooth surface. In this whole, cariogenic bacteria produce acids responsible for the decrease of pH, starting the process of demineralization [3].

Many products are being developed for caries control. The first was fluoride with remineralizing action, followed by chlorhexidine with antimicrobial activity, among others. The vehicle for these substances varies according to its clinical applications and can be found as rinses, gels, or varnishes used as nonsurgical methods for treating and preventing dental caries [4, 5].

Varnishes are preparations that differ in the polymeric matrix, pharmaceutical additives, and therapeutical agents, generally fluoride and chlorhexidine [6]. This ability to form a film takes place through a polymer, and among the most widely used are the ethyl cellulose and a mixture of copolymer and vinyl acetate-acrylate copolymers.

Recently, natural products are attracting the attention of several studies, mainly due to the increase in bacterial resistance and side effects of the antibiotics most commonly used [7–9]. Moreover, propolis has low toxicity and has several biological activities that strengthens its employment in healthcare. [10]. Several studies have demonstrated the antimicrobial activity of propolis extracts against cariogenic microorganisms [10, 11].

Chitosan is a derivative of chitin, a natural compound that can be found in arthropod exoskeletons, shells of crustaceans, and insect cuticles. Industrially, it is obtained by alkaline hydrolysis of chitin [12]. Chitosan is a biocompatible and biodegradable polymer. Its positive charge combines to the cell wall of bacteria, promoting a bactericidal and bacteriostatic property to this material [13]. These properties, coupled with the ability to form a film and adhere to the tooth, make chitosan an ideal base for sustained drug release [14]. Thus, the objectives of this study were to develop a dental varnish containing Brazilian green propolis ethanolic extract at three different concentrations and to verify the antimicrobial properties compared with chlorhexidine, cytotoxicity, and sustained-release profile of this new product.

## 2. Material and Methods

### 2.1. Propolis Sample and Propolis Extract Preparation

Propolis samples produced by honeybees (*Apis mellifera*) were collected during the spring and obtained in a beekeeping in Caeté, Minas Gerais, Brazil. Brazilian green propolis extract (EPE) was prepared according to Wojtyczka et al. [15]. Propolis was subjected to 14 days of extraction in order to obtain its ethanolic extract, which was later dissolved in 70% ethanol to obtain a 100 mg/mL working concentration. Briefly, the samples were ground mechanically and bottled in 10 g portions. The 10 g portions were put into flask and 100 g of 70% ethanol (w/v) was added. The flask was placed on a rotary shaker in a dark, closed room for two weeks at room temperature. After this period, the extract was cooled at 4°C for 24 h in order to precipitate all insoluble particles, which were removed from the propolis extract by filtration through filter paper (Whatman number 4). Next, the obtained filtrate evaporated to dryness at 40°C using a rotary vacuum evaporator. In order to prepare a working concentration, the brown colored viscous substance was dissolved in 70% ethanol. The propolis used has the main markers that give great quality to it [16, 17].

### 2.2. Propolis-Chitosan Varnish Preparation

Propolis varnishes were prepared by the addition of acetic acid to the EPE with or without dilution in ethanol. After mixing, chitosan (Sigma-Aldrich, St. Louis, USA) was added and Milli-Q (Millipore, Billerica, USA) water was used to complete the formulation volume. Propolis ethanolic extract was added and the compositions were mixed overnight to obtain the 5% (PV1), 10% (PV2), and 15% (PV3) propolis-chitosan formulations (Table 1). For being an innovative dental material, the request of the patent was registered at the National Institute of Industrial Property (INPI) under number 014100004357.

### 2.3. Antimicrobial Assay

Bacterial sensitivity or resistance to varnishes was detected by the disk diffusion assay, also known as the Kirby-Bauer method [18]. Aliquots of* Streptococcus mutans *(ATCC 70069),* Streptococcus sanguinis *(ATCC 10557),* Streptococcus salivarius* (INCQS-Oswaldo Cruz Foundation, Rio de Janeiro, Brazil/00457), and* Lactobacillus casei* (LC) (ATCC 393) containing 1.0 × 10^8^ CFU/mL were subcultured in agar Mueller-Hinton (Difco, Trenton, USA), supplemented with 5% of dextrose for* Streptococcus* spp.* L. casei* were subcultured and placed in Rogosa medium. Sterile filter papers soaked with 20 *μ*L of each propolis varnish were placed onto the agar. The controls were blank varnish/chitosan (CHV), PV1, PV2, PV3, and chlorhexidine varnish (VCX) (Fórmula e Ação, São Paulo, Brazil). The diameter of inhibition zone around the filter paper formed after 24 and 48 hours at 37°C in an atmosphere of 5% CO_2_ was measured in mm and recorded (M ± SD). Any inhibition zone around the filter paper measuring ≤7 mm was considered a negative result.

Minimal inhibitory concentration (MIC) test was carried out using tissue culture microplates (96 wells) containing 100 *μ*L/well BHI. For being highly viscous, the propolis varnish was diluted in an ethanol/water solution at 20% in a proportion of 1 : 1 (75 mg/mL). After being transferred to the first well, serial dilutions were performed to obtain concentrations ranging from 75 to 0,1 mg/mL. Chlorhexidine at 0.12% (Sigma-Aldrich, St. Louis, MO, USA) was used as positive control and BHI as negative control. The bacterial inoculum (1 × 10^6^ CFU/mL) was added to all wells, and the plates were incubated at 37°C in 5% CO_2_ for 24 hours. MIC was defined as the lowest concentration of the propolis varnish that inhibited microorganism visible growth indicated by resazurin 0.01% (Sigma-Aldrich, St. Louis, MO, USA). To determine minimal bactericidal concentration (MBC), an aliquot of each incubated well with concentrations higher than MIC was subcultured on BHI medium. MBC was defined as the lowest concentration of the propolis varnish that allowed no visible growth on the test medium [19].

### 2.4. Osteoblast-Like Cell Culture

Osteoblasts cells were donated from the Laboratory of Biomaterials and Molecular Entrapment (LEMB), Chemical Department, UFMG. This experiment was approved by the Ethics Committee on Animal Use of Federal University of Minas Gerais (CEUA number 167/2007). Osteoblasts were isolated by collagenase digestion of 20-day fetal rat calvariae [20]. Calvariae were dissected aseptically, and the frontal and parietal bones were stripped of their periosteum. Only the central portions of the bones, free from suture tissue, were collected. The calvariae were treated twice with phosphate-buffered saline (PBS) containing 4 mM EDTA (pH 7.4) for 15 min at 37°C in a shaking water bath. After being washed once in PBS, the calvariae were treated twice with 3 mL of 1 mg/mL collagenase for 7 min at 37°C. After the supernatants from these two digestions were discarded, the calvariae were treated two more times with 3 mL of 2 mg/mL collagenase (30 min, 37°C). The supernatants of the latter two digestions were pooled and centrifuged, and the cells were washed in Dulbecco's modified Eagle's medium (DMEM) with 10% fetal calf serum (FCS), suspended in further DMEM-10% FCS, and placed in 75 cm^2^ flasks. After 48 h, the media were changed to minimal essential medium (MEM) with 10% FCS. Confluence was reached within 5-6 days, at which time the cells were subcultured. After trypsinization with trypsin-EDTA (0.05%/0.53 mM), the cells were rinsed in MEM with 5% FCS, resuspended in fresh medium, and then seeded at 5 × 10^4^ cells/mL in 24-well plates (0.5 mL cell suspension/well, i.e., 2.5 × 10^4^ cells/well). The cells were incubated under 5% CO_2_-95% air at 37°C.

### 2.5. Cytotoxicity Assay

The cytotoxicity assay with osteoblasts was carried out according to ISO 10993-5 for cytotoxicity tests* in vitro*, using MTT colorimetric assay. The cells were cultured at 37°C in a humidified atmosphere of 95% air and 5% CO_2_ in DMEM (Cultilab, Campinas, Brazil) supplemented with 10% fetal bovine serum containing penicillin (10 IU mL^−1^) and streptomycin (10 mg mL^−1^). Thereafter, in each well 150 × 10^3^ cells were plated. Six wells were prepared for each varnish formulation and three for the control groups. The controls were lauryl sodium sulfate (LSS) (Sigma-Aldrich, St. Louis, USA) diluted in Milli-Q water in concentrations of 0.10%, 0.075%, 0.05%, and 0.025% and plates containing just cells. Plates were incubated at 37°C in 5% CO_2_ and 95% humidity conditions. The colorimetric assay was carried out after 24 hours and submitted to absorbance reading at 570 nm in spectrophotometer (Thermo Scientific Multiskan Spectrum; Thermo Fisher Scientific Inc., Boston, USA).

### 2.6. Sustained-Release Test

Bovine teeth were obtained from carcasses of animals that would be incinerated. The animals were sacrificed at specific slaughterhouse approved by the city of Belo Horizonte to market beef. The teeth were donated by the slaughterhouse before carcasses incineration. For the sustained-release test, ten incisors crowns of bovine teeth obtained* post-mortem* were cut into four pieces with diamond bur (KG Sorensen, São Paulo, Brazil). Each varnish formulation was applied to the buccal surface of each fragment, using five of them for each varnish and one fragment for CHV in each group. After applying and drying 40 *μ*L of the varnish, each fragment was placed into a tube with 1 mL of 20% ethanol/water solution and incubated in a shaker (KS4000 icontrol, IKA, Staufen, Germany) at 37°C and 30 rpm and after 0.5, 1.0, 2.0, 3.0, 4.0, 5.0, 6.0, 7.0, 8.0, 24, 72, 168.0, 336.0, 504.0, 672.0, 840.0, 1008.0, 1176.0, 1344.0, 1512.0, and 1680.0 hours, the solution was removed; a new one was added. Each sample of the solution (240 *μ*L) was placed in plates with 96 wells, adding 10 *μ*L of aluminum chloride solution [21]. The reading was carried out in spectrophotometer plate reader in an absorbance of 425 nm. BPE was used as positive control.

### 2.7. Statistical Analysis

**Figure 1:**
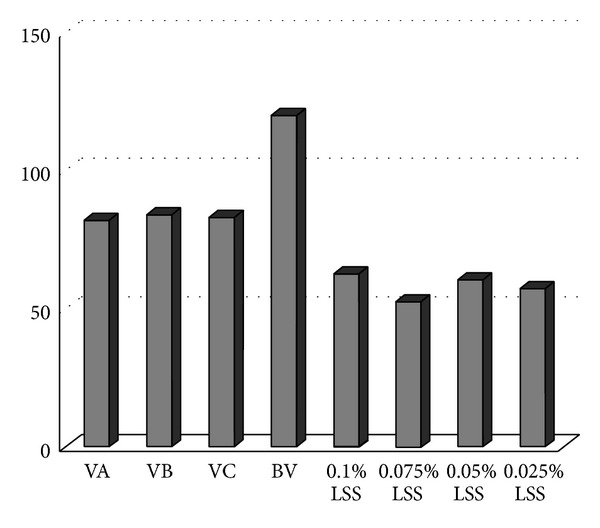
Viable cells (%) after 24 hours in contact with propolis varnishes and blank varnish from left to right: VA, VB, VC, BV, 0.1% LSS, 0.075% LSS, 0.05% LSS, and 0.025% LSS (percentage of viable cells/varnishes and control). VA = PV1 (5%); VB = PV2 (10%); VC = PV3 (15%); BV = CHV (chitosan varnish); LSS = lauryl sodium sulfate.

To determine whether the difference of the measures of inhibition zones was significant, data were statistically analyzed through Kruskall-Wallis nonparametric test, performed using SPSS for Windows v.17 (IBM Inc., Chicago, USA). In the cytotoxicity assay, absorbance results were converted into cell viability percentages and statistically compared by one-way ANOVA and Bonferroni test (Figure 1), using Graphpad Prism 5 (GraphPad Software Inc., San Diego, USA). *P* values lower than 0.05 were considered significant. The sustained-release profiles were compared using the difference factor and the similarity factor, according to the “independent model approach” [22]. Triplicates from at least three separated experiments were conducted in each assay.

## 3. Results

### 3.1. Antimicrobial Assay

All varnishes containing EPE inhibited* S. mutans*,* S. sanguinis*,* S. salivarius*, and* L. casei*.  Table 3 shows results of mean and standard deviation (M ± SD) for all tested formulations. No significant difference between the inhibition zones of the three tested concentrations of propolis (PV1, PV2, and PV3) and pure propolis extract (EPE) was observed. This demonstrates that propolis maintains its antimicrobial properties even when incorporated into the coating of chitosan. In contrast, chlorhexidine (CHX) showed significantly smaller areas than those observed in PV1, PV2, PV3, and EPE inhibition.* L. casei* appears to be more resistant to chlorhexidine than other microorganisms. The base coating of chitosan (CHV) showed significantly smaller inhibition areas for all microorganisms when compared with CHX and varnishes containing propolis.

MIC and MBC values for all products tested are shown in Table 3. MIC and MBC values ranged from 0,6 to 1,2 mg/mL for propolis varnish and 0,4 to 0,8 mg/mL for chlorhexidine (Table 3).

### 3.2. Cytotoxicity Test

Figure 1 shows the results of the cytotoxicity assay concentrations of propolis incorporated chitosan (VA = PV3 = 15%/VB = PV2 = 10%/VC = PV1 = 5%) compared with the chitosan varnish (BV = CHV = 15%) and sodium lauryl sulfate (SLS) at four different concentrations. Cell viability was greater in tests with CHV (≥100% of cells). Cell viability was similar for the three different concentrations of propolis (≥80%). Cytotoxicity was observed for all concentrations of SLS, which showed cell viability below 60%. This demonstrates that the varnish containing propolis and chitosan is not cytotoxic at the tested concentrations. These results confer low cytotoxicity, according to ISO 10993-5 standards.

### 3.3. Sustained-Release Test

**Figure 2:**
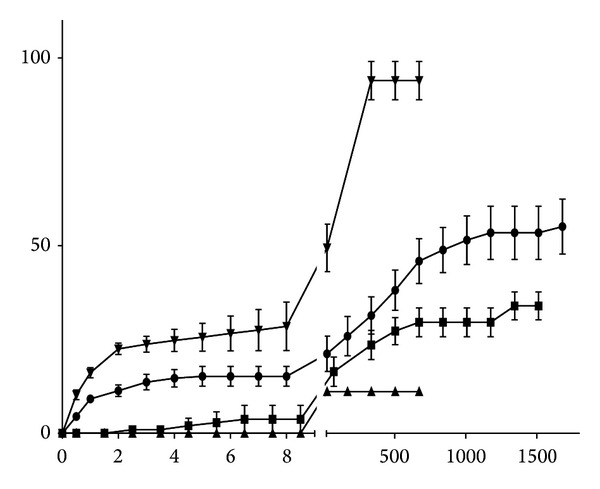
Slow-release profile of varnishes A (●), B (■), C (▲), and BGPEE (▼) (percentage of release/hours). A = PV3 (15%), B = PV2 (10%), C = PV1 (5%), and EPE = propolis ethanolic extract.

The extended-release profile considered the release of total flavonoids as quercetin. It was very heterogeneous as the varnishes were more viscous or more fluid depending on the concentration of propolis. Almost 100% of total quercetin from EPE was released in 24 hours. Varnish PV3 (15%) designed a release curve of 20% during the first 8 hours, thereafter stopping and restarting after 24 hours, becoming steady for 8 weeks (Figure 2). In the first two hours of the experiment, there was no release of PV2 (10%), starting just before this period and paralyzing after 7 hours. This inactivity lasted 72 hours and after this time the release started again. In this varnish only 30% of total quercetin was released in three weeks. The release of quercetin in PV1 (5%) started after 8 hours and only 10% of total flavonoids as quercetin were released within 24 hours.

## 4. Discussion

Propolis has been studied due to various biological activities highlighting its antimicrobial activity [11, 23, 24]. The results of antimicrobial susceptibility testing on* S. mutans* and* S. sanguinis* showed that propolis, even when associated with the varnish, is released in a satisfactory way, keeping its antimicrobial property, which makes the use of the drug feasible for this purpose (Table 2) [25]. All formulations inhibited the growth of all bacteria tested to a greater or lesser extent. This difference in size of inhibition zones between the formulations may be due to the concentration of EPE. This may be related to the molecular profile of propolis that has a variety of chemical compounds with different physicochemical characteristics, especially when comparing the inhibition observed for chlorhexidine, which has a characteristic solubility and molecular pattern that allows a better diffusion in agar.

Our results corroborate other studies of antimicrobial susceptibility of the ethanol extract of green propolis with* S. mutans*,* S. sanguinis*,* S. salivarius*, and* L. casei in vitro*, showing high activity of this product [10, 11, 26].

Chitosan varnish (CHV) showed low antimicrobial activity when compared with the other products. However, synergism between propolis and chitosan is possible to occur. The antimicrobial activity of chitosan was reported by several studies [27, 28] and this lack of activity may be due to the medium molecular weight of chitosan used in the varnishes, which is not soluble in water, unlike the low molecular weight of chitosan used in other studies.

Chitosan is a nontoxic, biocompatible, and chemically versatile polysaccharide. These properties enable this material to be used in drug delivery systems and tissue engineering, a promising tool in health care [29]. Its toxicity depends on the degree of deacetylation of chitin and its molecular weight. Nevertheless, most derivatives of chitosan have low toxicity [29], what might be seen in the cytotoxicity assay performed with osteoblasts in this study. BV, besides having not shown toxicity, allowed cell proliferation, increasing the amount in 20%.

Other studies showed that propolis ethanolic extract is cytotoxic on pulp fibroblasts [30] and cancer cells [31]. In the concentrations tested in this study, the cytotoxicity of the varnish containing propolis was considered low.

In the slow-release test, the components of propolis varnish were not soluble in artificial saliva, and as we could not quantify total flavonoids in this medium, we used an ethanol/water solution. As it does not simulate the oral environment, this test was just an indicative of the release.

The release of PV3 (15%), which has higher amount of EPE, remained stable in the early hours of the experiment, allowing a more constant release, which would ensure an effective and prolonged antimicrobial activity when applied clinically, relevant characteristics for the control of cariogenic biofilm.

For not having released quercetin in the first two hours of the experiment and not keeping regularity, CHV might allow the proliferation of microorganisms during the period of inactivity, although it presented satisfactory results in the antimicrobial susceptibility testing. For these reasons, their release profile might not represent an antimicrobial activity as effective in clinical practice.

PV1 (5%) released no quercetin during the first 8 hours of testing, what could allow bacterial growth in this period. Furthermore, the release of only 10% of quercetin in the oral environment and in just 24 hours limit the indication for the purpose it was developed. The similarity in the release profiles of formulations was compared by the “independent model approach.” In general, values lower than 15% (0–15%) and *F*_2_ values higher than 50% (50–100%) show the similarity of the sustained-release profiles [21]. None of the pair of formulations showed *F*_1_ lower than 15% or *F*_2_ higher than 50%, suggesting that all the release profiles are different from each other.

An* in vitro* study evaluated the release of chitosan containing dexamethasone and concluded this polymer allowed the slow-release of the drug tested [32]. Almost 90% was released within the first eight hours of experiment, result not obtained with any other varnish tested. The different characteristics of therapeutic agents used justify these differences.

Varnish formulations must release propolis right after being applied, remaining for about 24 hours onto the tooth surface, sustaining the release of the active principle on a regular and continuous basis, to achieve antimicrobial activity. However,* in vitro* tests may not reflect the* in vivo* responses, considering the environmental factors of the oral cavity and the genetic and social characteristics of each individual. Also, the product proved to be innocuous when tested in osteoblasts.

The concentration of 15% (PV3) has presented the largest inhibition zones and releases of higher profile, deserving further studies to prove its effectiveness.

## 5. Conclusions

Varnish preparations developed in this study showed satisfactory antimicrobial activity against* Streptococcus mutans*,* Streptococcus sanguinis*,* Streptococcus salivarius*, and* Lactobacillus casei* and demonstrated low cytotoxicity on osteoblasts (<50%). Varnish PV3 had the best results, deserving further studies to confirm its possible clinical efficacy.

## Figures and Tables

**Table 1:** Components of the varnish formulations PV1 (5%), PV2 (10%), and PV3 (15%).

Components	Product amount		
	PV1 (5%)	PV2 (10%)	PV3 (15%)
EPE	20.0 mL	40.0 mL	60.0 mL
Ethanol P.A.	40.0 mL	20.0 mL	No
Acetic acid	9.0 mL	9.0 mL	9.0 mL
Chitosan	1.0 g	1.0 g	1.0 g
Milli-Q water q.s.p.	100 mL	100 mL	100 mL

EPE (propolis ethanolic extract); PV (propolis varnish).

**Table 2:** Susceptibility test of propolis-based chitosan varnish against cariogenic bacteria; inhibition zones; mean and standard deviation (M ± SD) of three experiments.

Microorganisms	Inhibition zones (M ± SD) = mm					
	PV1 (5%)	PV2 (10%)	PV3 (15%)	CHV	EPE	CHX
*S. mutans* (ATCC 25175)	20.3 ± 0.51	20.2 ± 0.88	21.0 ± 0.00	10.4 ± 0.22*	22.6 ± 0.66*	19.0 ± 0.00
*S. sanguinis* (ATCC 10557)	21.5 ± 0.25*	21.5 ± 0.25*	22.3 ± 1.18*	10.5 ± 0.25*	21.3 ± 0.31*	19.0 ± 0.00
*S. salivarius * (INCQS 00457)	20.5 ± 0.33	20.5 ± 0.33	21.5 ± 0.50*	8.30 ± 0.33*	20.5 ± 0.55*	18.5 ± 0.55
*L. casei* (ATCC 393)	19.3 ± 0.25*	19.3 ± 0.25*	21.3 ± 0.71*	9.50 ± 0.55*	16.0 ± 0.00	17.3 ± 0.33*

PV: propolis-based chitosan varnish; CHV: chitosan varnish; EPE: propolis ethanolic extract; CHX: chlorhexidine 0.12%; INCQS: Instituto Nacional de Controle de Qualidade (National Institute of Quality Control, FIOCRUZ, Rio de Janeiro, Brazil).

*Are related to the statistical difference between the results (*P* < 0.05).

**Table 3:** MIC and MBC values from propolis varnish and positive control.

Product	MIC (mg/mL)	MBC (mg/mL)
Varnish 1 : 1	0.6–1.2	0.6–1.2
Clorhexidine (+control)	0.4–0.8	3.91–7.81

## References

[1] Petersen PE (2003). The world oral health report 2003: continuous improvement of oral health in the 21st century-the approach of the WHO global health programme. *Community Dentistry and Oral Epidemiology*.

[2] Dye BA, Tan S, Smith V (2007). Trends in oral health status: united States, 1988–1994 and 1999–2004. *Vital and Health Statistics*.

[3] Bowen WH, Koo H (2011). Biology of *Streptococcus mutans* derived glucosyltransferases: role in extracellular matrix formation of cariogenic biofilms. *Caries Research*.

[4] Petersson LG, Magnusson K, Andersson H, Almquist B, Twetman S (2000). Effect of quarterly treatments with a chlorexidine and a fluoride varnish on approximal caries in caries-susceptible teenagers: a 3-year clinical study. *Caries Research*.

[5] Zhang Q, van Palenstein Helderman WH, Van’t Hof MA, Truin G-J (2006). Chlorhexidine varnish for preventing dental caries in children, adolescents and young adults: a systematic review. *European Journal of Oral Sciences*.

[6] Steinberg D, Moldovan M, Molukandov D (2001). Testing a degradable topical varnish of cetylpyridinium chloride in an experimental dental biofilm model. *Journal of Antimicrobial Chemotherapy*.

[7] Cunha BA (2001). Antibiotic side effects. *Medical Clinics of North America*.

[8] Normark BH, Normark S (2002). Evolution and spread of antibiotic resistance. *Journal of Internal Medicine*.

[9] Liberio SA, Pereira ALA, Dutra RP (2011). Antimicrobial activity against oral pathogens and immunomodulatory effects and toxicity of geopropolis produced by the stingless bee Melipona fasciculata Smith. *BMC Complementary and Alternative Medicine*.

[10] Libério SA, Pereira ALA, Araújo MJAM (2009). The potential use of propolis as a cariostatic agent and its actions on mutans group streptococci. *Journal of Ethnopharmacology*.

[11] Paula AMB, Gomes RT, Santiago WK, Dias RS, Cortés ME, Santos VR (2006). Susceptibility of oral pathogenic bacteria and fungi to brazilian green propolis extract. *Pharmacologyonline*.

[12] Van Der Mei HC, Engels E, De Vries J, Dijkstra RJB, Busscher HJ (2007). Chitosan adsorption to salivary pellicles. *European Journal of Oral Sciences*.

[13] Uysal T, Akkurt MD, Amasyali M (2011). Does a chitosan-containing dentifrice prevent demineralization around orthodontic brackets?. *Angle Orthodontist*.

[14] Liu H, Chen B, Mao Z, Gao C (2007). Chitosan nanoparticles for loading of toothpaste actives and adhesion on tooth analogs. *Journal of Applied Polymer Science*.

[15] Wojtyczka RD, Dziedzic A, Idzik D (2013). Susceptibility of *Staphylococcus aureus* clinical isolates to propolis extract alone or in combination with antimicrobial drugs. *Molecules*.

[16] Righi AA, Negri G, Salatino A (2013). Comparative chemistry of propolis from eight brazilian localities. *Evidence-Based Complementary and Alternative Medicine*.

[17] Sawaya ACHF, Barbosa da Silva Cunha I, Marcucci MC (2011). Analytical methods applied to diverse types of Brazilian propolis. *Chemistry Central Journal*.

[18] Fani M, Kohanteb JJ (2012). Inhibitory activity of Aloe vera gel on some clinically isolated cariogenic and periodontopathic bacteria. *Journal of Oral Science*.

[19] Galvão LC, Furletti VF, Bersan SM (2012). Antimicrobial activity of essential oils against *Streptococcus mutans* and their antiproliferative effects. *Evidence-Based Complementary and Alternative Medicine*.

[20] Cornish J, Callon KE, Lin CQ-X (1999). Trifluoroacetate, a contaminant in purified proteins, inhibits proliferation of osteoblasts and chondrocytes. *American Journal of Physiology—Endocrinology and Metabolism*.

[21] Funari CS, Ferro VO (2006). Análise de própolis. *Ciênc Tecnol Aliment*.

[22] Costa P, Sousa Lobo JM (2001). Modeling and comparison of dissolution profiles. *European Journal of Pharmaceutical Sciences*.

[23] Sforcin JM, Bankova V (2011). Propolis: is there a potential for the development of new drugs?. *Journal of Ethnopharmacology*.

[24] Bankova V (2005). Recent trends and important developments in propolis research. *Evidence-Based Complementary and Alternative Medicine*.

[25] Duailibe SADC, Gonçalves AG, Ahid FJM (2007). Effect of a propolis extract on *Streptococcus mutans* counts *in vivo*. *Journal of Applied Oral Science*.

[26] Ccahuana-Vásquez RA, Cury JA (2010). S. mutans biofilm model to evaluate antimicrobial substances and enamel demineralization. *Brazilian Oral Research*.

[27] Decker E-M, Von Ohle C, Weiger R, Wiech I, Brecx M (2005). A synergistic chlorhexidine/chitosan combination for improved antiplaque strategies. *Journal of Periodontal Research*.

[28] Fujiwara M, Hayashi Y, Ohara N (2004). Inhibitory effect of water-soluble chitosan on growth of *Streptococcus mutans*. *New Microbiologica*.

[29] Kean T, Thanou M (2010). Biodegradation, biodistribution and toxicity of chitosan. *Advanced Drug Delivery Reviews*.

[30] Al-Shaher A, Wallace J, Agarwal S, Bretz W, Baugh D (2004). Effect of propolis on human fibroblasts from the pulp and periodontal ligament. *Journal of Endodontics*.

[31] Li F, Awale S, Tezuka Y, Kadota S (2009). Cytotoxic constituents of propolis from Myanmar and their structure-activity relationship. *Biological and Pharmaceutical Bulletin*.

[32] Rodrigues LB, Leite HF, Yoshida MI, Saliba JB, Junior ASC, Faraco AAG (2009). *In vitro* release and characterization of chitosan films as dexamethasone carrier. *International Journal of Pharmaceutics*.

